# Compressive and Thermal Properties of Non-Structural Lightweight Concrete Containing Industrial Byproduct Aggregates

**DOI:** 10.3390/ma15114029

**Published:** 2022-06-06

**Authors:** Ilenia Farina, Ivan Moccia, Cinzia Salzano, Narinder Singh, Payam Sadrolodabaee, Francesco Colangelo

**Affiliations:** 1 INSTM Research Unit, University Parthenope of Naples, Centro Direzionale, Is. C4, 80143 Naples, Italy; francesco.colangelo@uniparthenope.it; 2 Department of Engineering, University Parthenope of Naples, Centro Direzionale, Is. C4, 80143 Naples, Italy; ivan.moccia001@studenti.uniparthenope.it (I.M.); cinzia.salzano001@studenti.uniparthenope.it (C.S.); 3 Department of Civil Engineering, University of Salerno, 84084 Fisciano, Italy; snarinder@unisa.it; 4 Department of Civil and Environmental Engineering, Polytechnic University of Catalonia-BarcelonaTECH, 08034 Barcelona, Spain; payam.sadrolodabaee@upc.edu

**Keywords:** industrial waste, recycled artificial aggregates, lightweight concrete, circular economy, recycled waste PET, MSWI fly ash

## Abstract

This study aimed to investigate the recycling opportunities for industrial byproducts and their contribution to innovative concrete manufacturing processes. The attention was mainly focused on municipal solid waste incineration fly ash (MSWI-FA) and its employment, after a washing pre-treatment, as the main component in artificially manufactured aggregates containing cement and ground granulated blast furnace slag (GGBFS) in different percentages. The produced aggregates were used to produce lightweight concrete (LWC) containing both artificial aggregates only and artificial aggregates mixed with a relatively small percentage of recycled polyethylene terephthalate (PET) in the sand form. Thereby, the possibility of producing concrete with good mechanical properties and enhanced thermal properties was investigated through effective PET reuse with beneficial impacts on the thermal insulation of structures. Based on the obtained results, the samples containing artificial aggregates had lower compressive strength (up to 30%) but better thermal performance (up to 25%) with respect to the reference sample made from natural aggregates. Moreover, substituting 10% of recycled aggregates with PET led to a greater reduction in resistance while improving the thermal conductivity. This type of concrete could improve the economic and environmental aspects by incorporating industrial wastes—mainly fly ash—thereby lowering the use of cement, which would lead to a reduction in CO_2_ emissions.

## 1. Introduction

The building sector is one of the leading consumers of energy and natural resources, in addition to being one of the main contributors to CO_2_ emissions [1]. Therefore, producing more environmentally friendly materials obtained with sustainable processes by implementing the circular economy principles could be an interesting proposition, especially for concrete and cementitious materials [2]. In this context, there have been several recent studies in the literature regarding the use of waste and recycled materials in concrete as artificial aggregates [3,4,5,6] or fibers [7,8,9,10]; however, more novel and advanced solutions will still be needed to promote industrial symbiosis.

The amount of municipal solid waste (MSW) in the world is increasing day by day, and it is estimated to be roughly 3.4 billion tonnes by 2050 [11], which poses a serious problem for safe and efficient disposal [12]. One of the typical treatments applied to MSW is incineration, which significantly reduces the amount and volume of this kind of waste [13]. However, the residues of municipal solid waste incineration (MSWI), namely, fly ash (FA), need further treatment prior to landfilling because they contain contaminant elements [14]. Several studies have recently been conducted to investigate the possibilities of using industrial waste from MSWI in building materials as a treatment to immobilize the contaminant elements [15,16]. The outcomes have permitted us to conclude that fly ash (FA) is suitable for several applications in the construction sector [17,18,19]. MSWI-FA use in the production of lightweight concrete blocks is one of the most effective methods for reducing environmental impacts [20,21,22,23]. 

Considering that the global aggregate production surpasses 40 billion tonnes annually [24], using recycled and artificial aggregates as a replacement for natural ones is considered among the most effective strategies for more sustainable concrete. In this regard, several studies have already investigated the incorporation of industrial byproducts (specifically, fly ash from coal plants) [25,26,27], as well as the construction and demolition wastes (CDWs) [28,29,30,31,32], as lightweight aggregates (LWAs) in concrete. Lightweight artificial pellets from byproducts are typically produced and hardened through sintering (the most common method, but it is energy intensive), autoclaving, or cold-bonding processes [33,34]. 

Lightweight concretes have a lower density than conventional concretes, thanks to a system of voids in the matrix that replace natural aggregates in part or completely with LWA [35]. The latter has a lower average density than that of normal aggregates [36,37,38], and the corresponding concrete is identified as “concrete with light aggregates” or simply “light concrete” [39]. However, the open porosity and water absorption of the recycled aggregates are typically higher than those of the natural ones, which may lead to adverse impacts on the strength, drying shrinkage, and durability of the manufactured concrete [40,41]. Thus, some methods, including CO_2_ curing (accelerated carbonation) of recycled aggregates or the addition of pozzolanic micropowders, can offset the mentioned drawbacks and enhance the physical and mechanical characteristics of the artificial aggregates [42,43].

The use of lightweight concrete allows for excellent performance in terms of fire resistance and reduced weight for the structure, along with saving costs [44,45]. For this reason, they are often employed in the renovation or construction of elevations or buildings in seismic areas [46]. The employment of lightweight concrete also leads to economic benefits because of the decreased permanent loads on the structure [47]. The reduced weight of the structure also allows one to build on the less load-bearing ground without resorting to the complex and expensive foundations, while, above all, guaranteeing the same pressures transmitted to the ground to build buildings with a greater vertical development [48,49,50]. Lightweight concrete has a higher ductility than that of ordinary concrete, which is required for anti-seismic structures; the greater the ductility of the material, the greater the capacity to dissipate energy before collapsing [51,52].

In recent years, there has also been a significant increase in the use of lightweight concrete blocks to improve thermal insulation [53]. It has been observed that lightweight concrete (LWC) is a good material for the thermal insulation of structures [54]. In order to obtain buildings with good thermal insulation performance, the addition of plastic materials to concrete has been taken into consideration [55]. Among all of the plastic materials, polyethylene terephthalate (PET) is one of the main recycled materials used to perform thermal insulation in buildings [56]. Many authors have already investigated the suitability of plastic waste in cement and/or concrete [57,58]. It is clear that the use of this type of waste in the construction field may represent an effective solution for reducing the environmental impact of plastics and, thus, to contribute to the development of an increasingly sustainable building industry.

Using recycled PET waste as an aggregate in Portland cement concrete/mortar in addition to geopolymer ones was recently studied. For instance, Akçaözoǧlu et al. [59] investigated the replacement of PET with conventional aggregates in the range of 30–60% in Portland cement concrete, while in another study [60], the usage of more than 60% PET in alkali-activated mortars was evaluated. All of these research works concluded that the compressive and flexural strengths of the concrete were reduced by the addition of PET waste, which was mainly due to the weak adherence between PET granules and the paste. Moreover, the addition of PET caused a rise in the porosity of the mixtures due to its low density. Nonetheless, it was reported that a small substitution of PET (5%) marginally decreased the compressive and split tensile strengths (around 2%) [61], which indicated that the reduction in strength happened proportionally to the addition of PET [62]. Further, adding 75% PET could increase the workability of the concrete by 100% with respect to the normal concrete due to its spherical and smooth shape.

While some types of LWAs, mainly from CDW, have already become standardized according to EN13055 [63] and ASTM C330M-C332M [64,65,66], further research is still needed on LWAs produced through more novel processes, such as cold-bound or recycling products, including PET waste, to identify the projected applications [67]. Generally, LWAs can be used both in lightweight structural concretes and non-structural ones—such as in masonry or insulation—based on the final properties of the produced concrete. According to ASTM C330M [64], concrete with 100% LWAs may be used in structural-oriented applications if the 28-day compressive and splitting tensile strengths are at least 17.0 and 2.0 Mpa, respectively, and the density is not higher than 1600 Kg/m^3^. Nonetheless, there are still uncertainties about the quality of recycled aggregated concretes, and thus, the majority of standards and specifications recommend replacing up to 30% of recycled aggregates in structural elements [40]. 

In view of what was mentioned above, further studies are still needed to promote the use of recycled aggregates obtained from wastes and byproducts with a higher percentage in cementitious products. While the majority of studies in the literature focus on the use of recycled aggregates from CDW and/or energy-intensive sintered and pulverized ashes/slags, in this study, the concrete includes artificial LWAs from the cold-bonding technique; PET sand was produced, and its mechanical and thermal properties were characterized. To this end, in the first part of this research work, washed MSWI fly ash (FA) was used together with GGBFS to replace cement in a properly evaluated mix design in order to perform a cold-bonding granulation process and obtain recycled aggregates. A further granulation process was carried out on the produced aggregates to improve the stabilization/solidification process, as described in depth in [68]. The latter was used as a complete replacement in concrete samples in order to evaluate their properties by comparing them to those of a standard concrete sample. In addition, a further substitution of aggregates was made by using recycled PET. Thus, the aim of this study is to optimize the waste addition in terms of the physical, mechanical, and thermal performance of the resulting concrete in light of the synergistic actions of both industrial pelletized byproducts and plastic materials.

## 2. Materials and Methods

### 2.1. Materials

#### 2.1.1. Lightweight Artificial Aggregates

The chemical compositions of the main materials, including municipal solid waste incineration fly ash (MSWI-FA), ground granulated blast furnace slag (GGBFS), marble sludge (MS), and cement (CEM II/A-L 42.5R), used to perform the cold bonding granulation process are gathered in Table 1. The FA was washed before usage, as described in [69], to reduce the amounts of unfavorable metals. The artificial aggregates were produced by using a disk granulator device through the cold-bond process described in [1], with the amounts shown in Table 2.

**Figure 1:**
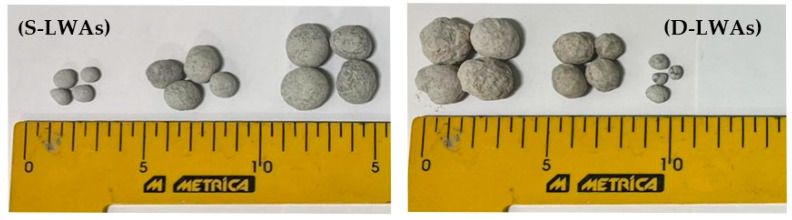
Single lightweight artificial aggregates (S-LWAs) and double lightweight artificial aggregates (D-LWAs).

The produced single lightweight artificial aggregates (S-LWAs) (Figure 1) underwent 28 days of curing in a wet environment by means of a manual nebulizer to avoid dehydration and the subsequent breakout of the external surface. Half of the S-LWAs were used to perform a double-step cold-bonding granulation with the mix design reported in Table 3 for the production of double lightweight artificial aggregates (D-LWAs). As can be seen in Figure 1, the produced cold-bonded aggregates had a more rounded shape than an angular shape, which might have enhanced the workability while worsening the adherence with the mortar [40].

Table 4 gathered the physical properties of the manufactured aggregates, including their density, open porosity (OP), and water absorption capacity (WAC). The values belong to the three different diameter size fractions considered and represent the properties of the average diameter size for each diameter fraction. Likewise, Table 5 shows the properties of the natural aggregates used to produce the reference sample. 

As can be observed in Table 4, the Mix 1 granules containing 80% FA had a lower density on average, but a higher porosity and WAC with respect to the granules with less FA. A similar trend was observed by Ding et al. [70], where the increase in the amount of industrial waste in the cold-bonded aggregates from 60% to 90% decreased the density by 12% while increasing the WAC by 7%. In comparison with the recycled aggregates produced from CDW, according to Gomes et al. [42], the density of the cold-bonded aggregates produced in the present study was, on average, lower, whereas the WAC was higher. Nonetheless, the sintered coal FA pellets produced by Ramamurthy et al. [33] and Yang et al. [71] demonstrated higher WAC values, which were in the ranges of 15–22% and 21–33%, respectively. Further, the density of the granules produced from four types of sintered MSWI-FA by Mangialardi [72] was reported to be in the range of 2.17–2.5 g/cm^3^, which was higher than that in the present study, and they were considered to be normal-weight aggregates.

#### 2.1.2. Polyethylene Terephthalate

Polyethylene terephthalate (PET) is a member of the polyester family of polymers; it is mainly a thermoplastic resin made of phthalates [73]. Plastic waste causes major challenges and the development of issues due to the increasing requirement for plastic every day, the development of plastic companies, and the few available areas for disposal [74]. Unfortunately, PET recycling is lower than its actual usage; therefore, discovering innovative methods for maximum recycling of this material has become crucial. PET sand with a particle size of less than 6 mm and a bulk density of 300 kg/m^3^ was provided by the company Vedelago Recycling Center Ltd. (Treviso, Italy).

### 2.2. Methods 

#### 2.2.1. Concrete Sample Manufacturing

Recycled aggregates with single- and double-step granulation processes were produced, and the mechanical and thermal properties of concrete containing such aggregates were investigated. Several samples of cubic concrete with a side of 10 cm were manufactured. Complete substitution of aggregates was performed to manufacture three concrete mixtures using single lightweight aggregates (S-LWA samples) and three samples using double lightweight aggregates (D-LWA samples). Each mixture was cast in a set of 3 conventional concrete specimen molds. The percentage distribution of the aggregates was about 25% of 4–8 mm (fine) aggregates, 50% of 8–16 mm (medium) aggregates, and 25% of 16–20 mm (coarse) aggregates.

In order to obtain improved thermal properties, six further concrete mixtures were manufactured in the same way as the above-mentioned specimens, where 10% of the medium-diameter aggregates by volume were replaced with an equivalent quantity of PET (S-LWA+PET and D-LWA+PET). The 10% substitution was selected based on the results of a previous study [75]. Finally, a reference sample (REF) from a natural aggregate (NA) was manufactured for comparison. For the three REF specimens, the NA distribution was about 25% sand, 50% medium aggregate, and 25% coarse aggregate with the properties gathered in Table 5. Table 6 summarizes the component percentages of the manufactured concrete samples: S-LWC and D-LWC refer to concrete samples containing 100% single and double lightweight aggregates, respectively, while S-LWC+PET and D-LWC+PET refer to samples containing 10% PET as a fine aggregate. Finally, REF refers to the reference sample with 100% natural aggregates.

**Figure 2:**
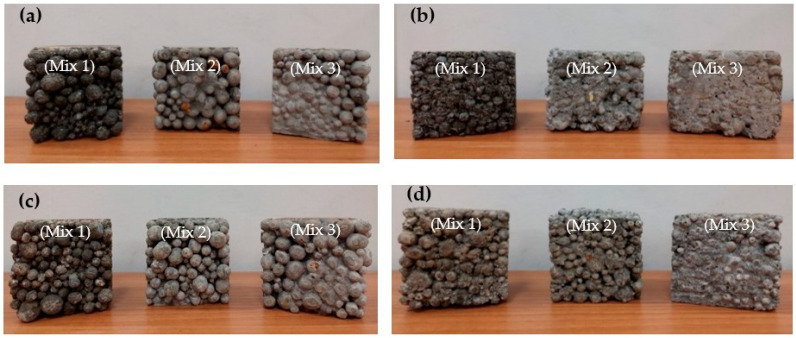
Lightweight concretes: (**a**) single lightweight concrete; (**b**) single lightweight concrete with PET; (**c**) double lightweight concrete; (**d**) double lightweight concrete with PET.

All of the sample sets were cast in cubic steel molds (10 cm × 10 cm × 10 cm) and then covered with cellophane film. After 24 h, they were removed from the molds and cured for 28 days at room temperature. Figure 2 shows some of the samples produced.

#### 2.2.2. Compressive Strength of Concrete Samples

**Figure 3:**
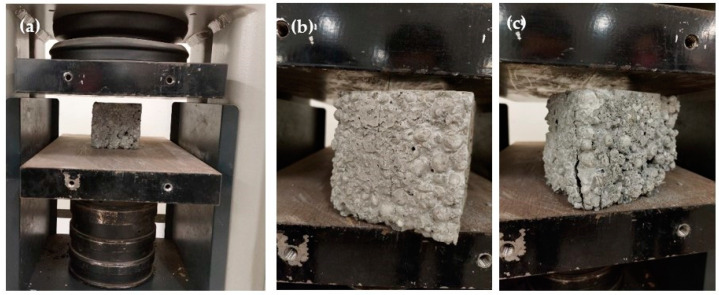
Experimental setup of the compressive test: (**a**) initiation of the test; (**b**) failure mode of D-LWC Mix2; (**c**) failure mode of D-LWC+PET Mix2.

Compressive strength is the ability of a material or structure to carry loads on its surface without any cracks or deflections. The tested sample, usually in the form of a cube, prism, or cylinder, is compressed between the plates of a compression-testing machine by a gradually applied load. While the vertical direction shortens, the horizontal one expands, and cracks occur increasingly until the sample fails. The compressive strength of the examined cubic samples after 28 days of curing was determined according to UNI EN 12390-3 [76] with a Controls MCC8 hydraulic console with a 2000 kN capacity, as shown in Figure 3a.

#### 2.2.3. Thermal Conductivity of Concrete Samples

**Figure 4:**
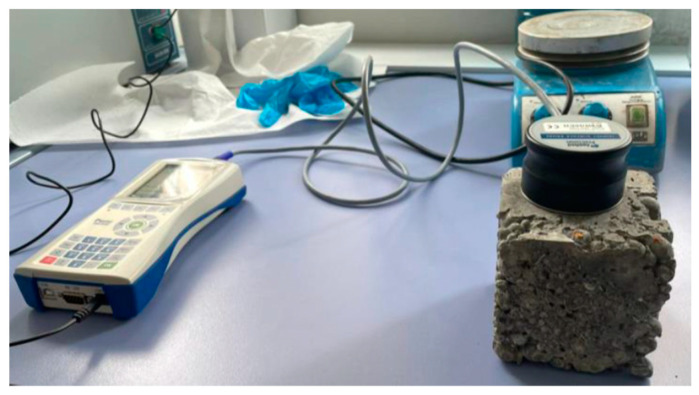
Experimental setup of the thermal conductivity test.

The thermal conductivity of the concrete samples was evaluated using an ISOMET 2114 portable thermal characterization analyzer (Figure 4), which was able to perform a relatively rapid test to evaluate the thermal characteristics thanks to a surface probe by means of a modified transient pulse method. This steady-state method, which is based on ASTM D5930 [77], was similarly used and reported in other studies [78]. The thermal conductivity coefficient was measured on a dried sample in a room-temperature environment with a relative humidity of about 50%. When the concrete specimen was in thermal equilibrium with the surrounding environment, the heat flow was generated by applying a heat impulse. Cube specimens with a side length of 100 mm were used to test the thermal properties. In order to exclude the effect of surface heterogeneity on the test results, the probe was placed in the center of the specimen’s surface. Two opposite surfaces of each sample were used for the test, and each surface was tested three times.

## 3. Results and Discussion 

### 3.1. Compressive Strength

The mean results of the compressive test evaluated for each mixture (three cubic specimens) are summarized in Table 7. As can be observed, the compressive strength of the concrete specimens with single-step artificial aggregates (S-LWC) produced with Mix1, Mix2, and Mix3 decreased by 42.13%, 36,64%, and 39.94%, respectively, compared to that of the REF sample. A similar trend was recorded for the D-LWC aggregates, with a decrease of 38.83% for Mix1, 31.54% for Mix2, and 34.23% for Mix3 compared to the NA concretes. According to the outcomes obtained, the highest percentage of FA used in the mixture resulted in the lowest compressive strength values, while the best values for the compressive strength were recorded for S-LWC Mix2 and D-LWC Mix2 with 75% FA. In other words, the concrete samples containing Mix1 aggregates that had 80% FA, which had the lowest density among the aggregates (see Table 2), led to the lowest concrete densities and, consequently, the lowest compressive strengths. A similar effect of aggregate density on the compressive strength of concrete was observed by Chen et al. [79] in a study on synthetic lightweight aggregate concretes from reservoir sediments. Baykal et al. [80] reported that concrete containing lightweight aggregates produced with cold-bonded coal FA had, on average, 30–40% lower compressive strength at 28 days than that of the reference sample; thus, the concrete could be proper for non-structural and semi-structural applications. Moreover, Kazemi et al. [81] reported that the incorporation of recycled aggregates from CDW caused a great reduction (up to 35%) in the compressive strength of the reference sample, which was consistent with the present study. Further, by reviewing several studies, Salgado and Silva [40] concluded that the compressive resistance of concrete was reduced as the ratio of recycled aggregates increased. Nonetheless, the sustainability and environmental impacts were enhanced by increasing the percentage of recycled aggregates in the concrete [30].

**Figure 5:**
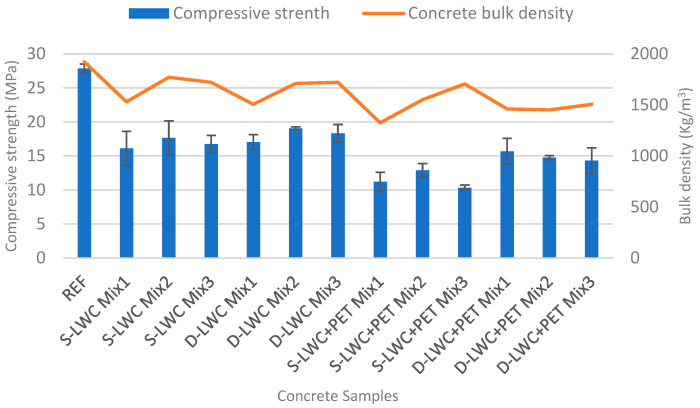
Effect of concrete density on the compressive strength.

As can be observed in Figure 5, the strengths recorded for the concrete specimens with 10% PET replacement showed significant reductions of 59.81% for S-LWC+PET Mix1, 53.70% for S-LWC+PET Mix2, and 62.90% for S-LWC+PET Mix3 compared to the REF concretes. The same samples also showed reductions in compressive strength values of 30.54%, 26.93%, and 38.22% for Mix1, Mix2, and Mix3, respectively, compared to the S-LWC samples. In comparison to the NA concretes, there were significant reductions of 43.68% for the D-LWC+PET Mix1 concrete with a 10% PET volumetric replacement ratio, 46.98% for D-LWC+PET Mix2, and 48.60% for D-LWC+PET Mix3. Comparing the compressive strength values obtained for D-LWC+PET to the those of the respective concrete specimens without the addition of PET, resistance reductions of 7.93%, 22.56%, and 21.85% were recorded for D-LWC+PET Mix1, D-LWC+PET Mix2, and D-LWC+PET Mix3, respectively. 

The decreased values of the compressive strength for the concretes with a 10% PET volumetric replacement ratio were mainly due to the shape and flexibility of the PET flakes [82], as well as their low density. The addition of PET granules in the mixtures led to increased porosity, as well as a weak bond between the inorganic matrix and the organic PET granules [61], which resulted in lower compressive strength values, as similarly reported in other studies [83,84]. Colangelo et al. [85] observed that increasing the percentage of recycled plastic materials (polyolefins) as aggregates in concrete resulted in lower density, higher porosity, and, consequently, worsening of the mechanical performance. As can be observed in Figure 3, a deeper crack under compression appeared in the specimen that included PET due to the ineffective bond at the interfacial transition zone between the matrix and the aggregate. In fact, hydrophobic waste polymer aggregates could have an adverse influence on the hydration rate and could limit mechanical development [86].

As most of the lightweight aggregate concrete samples had a 28-day compressive strength of less than 17 Mpa; according to the American Concrete Institute (ACI) [87], they can be considered for non-structural applications. Only samples of double-layer aggregates without PET and S-LWC Mix2 had the minimum resistance required to be considered for structural applications. Further, based on ASTM C330M [64], the density of lightweight structural concrete should be less than 1600 kg/m^3^; thus only D-LWC Mix1 met both requirements of strength and density to be considered as lightweight structural concrete.

### 3.2. Thermal Conductivity 

**Figure 6:**
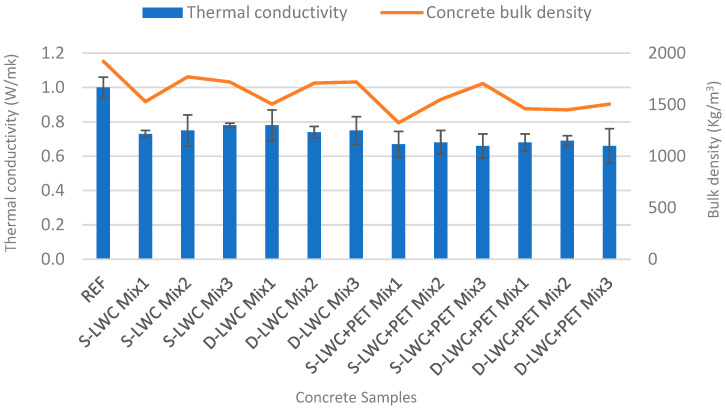
Effect of concrete density on the thermal conductivity.

The results of the thermal conductivity test are summarized in Table 7 and Figure 6. As can be seen, the thermal conductivity of the natural aggregate concrete sample was about 1.0 W/mK, and this was an expected value according to the UNI EN 10351 standard [88]. The comparison of the thermal conductivity values between the artificial aggregate concretes and the reference concrete highlighted that the use of recycled aggregates led to average reductions of conductivity of about 27% for the S-LWC1, 25% for the S-LWC2, and 22% for the S-LWC3 samples. Similar reductions of 22%, 26%, and 25% were recorded for the D-LWC1, D-LWC2, and D-LWC3 samples compared to the reference samples. In particular, the increase in FA content entailed a reduction in the thermal conductivity in the S-LWC Mix1 sample, which was related to the lower density of FA with respect to cement, resulting in more insulative concrete samples than conventional concrete, as also reported in [89]. As can be seen in Table 7, the density of the concrete for Mix1 decreased by more than 20% with respect to the REF sample, while for Mix2 and Mix3, this reduction was limited to 10%. The incorporation of the light aggregates resulted in a lowering of the density and unit weight of the concrete [75], which led to a decrease in thermal conductivity, as similarly reported in other studies [34,59].

Moreover, the addition of PET to the mixtures containing single- and double-step aggregates resulted in a further conductivity reduction compared to the control samples. It can be noted that the 10% addition of PET in the S-LWC+PET samples showed reductions of 33%, 32%, and 34% compared to the decreases of 32%, 31%, and 34% obtained for the D-LWC+PET samples with respect to the reference sample. In fact, the addition of PET increased the porosity of the material, thereby lowering density, and the porous cementitious materials had a lower thermal conductivity and sound absorption coefficient, as reported in other recent studies [78,90,91]. The density of the samples containing PET was reduced up to 15% on average in comparison with their counterparts without PET; see Table 7.

Considering both the mechanical and thermal behaviors, the best mixture was provided by the double-step pelletized D-LWC Mix2 concrete, which exhibited higher resistance and lower thermal conductivity with respect to D-LWC Mix1 and D-LWC Mix3. In fact, Mix2 containing double-step pelletized aggregates without PET addition could improve the thermal conductivity of the conventional concrete by 26%, while the compressive strength was still satisfactory (19.06 MPa). The differences between the aforementioned mixes were minimal in the case of PET addition (D-Mix2-PET and D-Mix3-PET samples), which surely provided a beneficial effect to both mixes by further reducing the overall thermal conductivity of the concrete samples. In this case, the best overall thermal conductivity attained was 34% less than that of conventional concrete. This outcome and the fact that the addition of PET reduced the compressive strength of the two samples, bringing them to similar values (14.76 and 14.31 MPa, respectively), were considered. Thus, the mix designs with low PET content (10%) were considered to enhance the thermal properties while preserving the mechanical characteristics of the produced concrete as much as possible. However, PET would not properly bind to cement, reducing the compressive strength of the manufactured concrete, i.e., all of the PET samples had resistances lower than 16 Mpa and were, therefore, considered as non-structural concrete.

## 4. Conclusions

The incorporation of recycled aggregates in construction products leads to less exploitation of natural aggregates and resources, as well as a reduction in the amount of waste disposed of in landfills. In this study, artificial aggregates made from industrial wastes—municipal solid waste incineration fly ash, slag, and marble sludge—were used to produce lightweight concrete (LWC) that could be oriented toward non-structural applications. A compression test on the LWC samples showed that the concrete had limited compressive strength and, on the other hand, a lower thermal conductivity when incorporating polyethylene terephthalate (PET) compared to that of its PET-free counterpart.

Specifically, the following conclusions are drawn from the results:

- Using artificial recycled aggregates with a 100% substitution resulted in a decrease of more than 30% in compressive resistance with respect to the reference sample (REF), which was composed of 100% natural aggregates. Moreover, adding 10% PET to the mixture resulted in an additional average 22% reduction in compressive resistance.
- The majority of the lightweight concrete samples had compressive resistances below 17 MPa, making them suitable for non-structural applications.
- The addition of PET could improve the thermal conductivity of the samples, on average, by 10% and 33% for the PET-free and REF samples, respectively. Thus, adding PET led to lower mechanical properties while improving the thermal properties due to an increase in the trapped air and porosity. The amount of PET could be increased for possible specific application in which a lower thermal conductivity and a lower strength are needed, such as in concrete panels for façade cladding or partition walls.
- The lightweight concrete sample composed of the double-step aggregates (with the mix design of 75% fly ash) without PET demonstrated the optimal performance when considering both thermal and compressive behaviors.

Therefore, using this type of artificial aggregate as a component of lightweight concrete can immobilize hazardous industrial waste (fly ash from municipal solid waste incineration) and promote a circular economy trend in the construction and building material industry. Future studies could employ CO_2_ curing in the production of this kind of aggregate, as well as the manufacturing of concrete samples with distinct replacement ratios of recycled aggregates.

## Figures and Tables

**Table 1:** Chemical compositions of the materials used for the production of aggregates (wt %).

Mix Design	MSWI-FA	Washed FA	GGBFS	MS	Cement
Fe_2_O_3_	1.43	1.39	0.3	1.35	3.41
CaO	24.69	42.97	43.9	51.92	67.16
CO	10.46	20.50	-	22.74	-
SiO_2_	5.01	6.25	35.7	14.16	16.65
Al_2_O_3_	2.11	4.43	11.2	4.56	4.21
SO_3_	7.87	9.07	-	-	5.34
MgO	1.28	2.32	6.5	1.21	1.71
SnO_2_	-	-	-	2.20	-
Na_2_O	14.57	4.84	0.8	0.86	-
K_2_O	7.20	1.87	-	1.02	1.54
TiO_2_	0.64	0.77	0.51	-	-
ClO	23.29	4.40	-	-	-
ZnO	1.45	1.19	-	-	-

**Table 2:** Mix design used for cold-bonding granulation.

Mix Design	FA (%)	GGBFS (%)	Cement (%)
Mix 1	80	5	15
Mix 2	75	10	15
Mix 3	70	15	15

**Table 3:** Mix design used for the double-step cold-bonding granulation.

Mix Design	MS (%)	GGBFS (%)	Cement (%)
	60	25	15

**Table 4:** Physical properties of the manufactured aggregates.

		S-LWA			D-LWA		
	Particle Size	Density [g/cm^3^]	OP [%]	WAC [%]	Density [g/cm^3^]	OP [%]	WAC [%]
Mix 1	4–8 mm	1.98	17.68	10.68	1.80	15.77	9.41
	8–16 mm	1.81	19.07	12.06	1.67	10.01	5.74
	16–20 mm	1.54	22.37	14.07	1.94	10.47	6.13
Mix 2	4–8 mm	2.14	7.66	3.94	1.85	10.88	5.79
	8–16 mm	1.89	6.33	3.31	1.73	10.17	5.44
	16–20 mm	1.60	5.80	3.18	1.57	7.34	3.79
Mix 3	4–8 mm	2.29	4.86	2.51	1.83	8.79	4.73
	8–16 mm	1.99	4.49	2.41	1.89	6.92	3.75
	16–20 mm	1.83	2.53	1.39	1.77	5.10	2.70

**Table 5:** Physical properties of the natural aggregates.

Particle Size	Density [g/cm^3^]	WAC [%]
Fine	2.45	2.15
Medium	2.70	1.65
Course	2.85	1.35

**Table 6:** Mix design for a concrete sample. (Note: S-LWC: single lightweight concrete; D-LWC: double lightweight concrete; S-LWC+PET: single lightweight concrete with 10% addition of PET; D-LWC+PET: double lightweight concrete with 10% addition of PET; REF: reference sample with natural aggregates).

	PET (cm^3^)	S-LWA (cm^3^)	D-LWA (cm^3^)	NA (cm^3^)	Cement (cm^3^)	Water (cm^3^)
S-LWC	-	1200	-	-	230	120
D-LWC	-	-	1200	-	230	120
S-LWC+PET	120	1080	-	-	230	120
D-LWC+PET	120	-	1080	-	230	120
REF	-	-	-	1200	230	120

**Table 7:** Average density, compressive strength, and thermal conductivity of the tested concrete samples.

Mixtures	Bulk Density (kg/m^3^)	Compressive Strength (MPa)	CoV of Compressive Strength (%)	Thermal Conductivity (W/mK)
REF	1921	27.84	3	1.00
S-LWC Mix1	1530	16.11	15	0.73
S-LWC Mix2	1770	17.64	14	0.75
S-LWC Mix3	1720	16.72	7	0.78
D-LWC Mix1	1505	17.03	6	0.78
D-LWC Mix2	1710	19.06	1	0.74
D-LWC Mix3	1720	18.31	7	0.75
S-LWC+PET Mix1	1325	11.19	12	0.67
S-LWC+PET Mix2	1550	12.89	7	0.68
S-LWC+PET Mix3	1705	10.33	3	0.66
D-LWC+PET Mix1	1460	15.68	12	0.68
D-LWC+PET Mix2	1450	14.76	2	0.69
D-LWC+PET Mix3	1505	14.31	13	0.66

Note: CoV stands for coefficient of variance.

## Data Availability

The data presented in this study are available upon request from the corresponding author.
